# Energy–entropy method using multiscale cell correlation to calculate binding free energies in the SAMPL8 host–guest challenge

**DOI:** 10.1007/s10822-021-00406-5

**Published:** 2021-07-15

**Authors:** Hafiz Saqib Ali, Arghya Chakravorty, Jas Kalayan, Samuel P. de Visser, Richard H. Henchman

**Affiliations:** 1 Manchester Institute of Biotechnology, The University of Manchester, 131 Princess Street, Manchester, M1 7DN UK; 2 Department of Chemistry, The University of Manchester, Oxford Road, Manchester, M13 9PL UK; 3 Department of Chemical Engineering and Analytical Science, The University of Manchester, Oxford Road, Manchester, M13 9PL UK; 4 Department of Chemistry, University of Michigan, Ann Arbor, MI 48109 USA; 5 Present Address: Sydney Medical School, The University of Sydney, Sydney, NSW 2006 Australia

**Keywords:** Host-guest binding, Free energy methods, Molecular dynamics simulation, Entropy

## Abstract

Free energy drives a wide range of molecular processes such as solvation, binding, chemical reactions and conformational change. Given the central importance of binding, a wide range of methods exist to calculate it, whether based on scoring functions, machine-learning, classical or electronic structure methods, alchemy, or explicit evaluation of energy and entropy. Here we present a new energy–entropy (EE) method to calculate the host–guest binding free energy directly from molecular dynamics (MD) simulation. Entropy is evaluated using Multiscale Cell Correlation (MCC) which uses force and torque covariance and contacts at two different length scales. The method is tested on a series of seven host–guest complexes in the SAMPL8 (Statistical Assessment of the Modeling of Proteins and Ligands) “Drugs of Abuse” Blind Challenge. The EE-MCC binding free energies are found to agree with experiment with an average error of 0.9 kcal mol^−1^. MCC makes clear the origin of the entropy changes, showing that the large loss of positional, orientational, and to a lesser extent conformational entropy of each binding guest is compensated for by a gain in orientational entropy of water released to bulk, combined with smaller decreases in vibrational entropy of the host, guest and contacting water.

**Supplementary Information:**

The online version contains supplementary material available at 10.1007/s10822-021-00406-5.

## Introduction

The accurate prediction of binding between molecules in solution is a key question in theoretical and computational chemistry. It has relevance to much of chemistry but also more broadly to fields such as biology, pharmacology, chemical engineering and environmental science. Under ambient conditions, binding is governed by the change in Gibbs free energy Δ*G* = − *RT* ln *K* where *RT* is the gas constant times temperature and *K* is the equilibrium constant, which is the ratio of probability of the bound form relative to the unbound form at equilibrium for a given concentration of the molecules involved, typically 1 M.

Many methods have been developed to calculate binding free energy, which feature the typical trade-off of speed versus accuracy [1–4]. At the faster end are scoring functions which are parametrised to reproduce known binding data, being made ever more accurate by using larger data sets and machine-learning methods that resolve the optimal model form at the cost of providing less molecular insight [5–10]. Simulation methods using classical potentials can determine the free energy difference from the relative probability of the bound and free states, whether this be by brute-force sampling, biased simulations such as metadynamics [11] or umbrella sampling [12, 13], or alchemical methods such as free energy perturbation [14, 15] or thermodynamic integration [16, 17] which utilise shorter, unphysical binding paths by varying the molecules’ interacting Hamiltonian rather than their positions. Combining these methods with more accurate electronic-structure methods is not yet achievable when simulating ensembles of solvated systems for multiple states along a path. However, they or regular force fields can be used in the energy–entropy (EE) class of methods which evaluate the free energy of the bound and free states separately and directly from the system energy and entropy and get the binding free energy from their difference. These are sometimes referred to as “end-point” methods in the context of a free energy difference between two states but can equally be applied to single state points with no specified end.

EE methods are more approximate and limited than other methods because calculating the entropy requires knowing the probability distribution of all quantum states of a system involving both solutes and solvent. This goes beyond the usual analyses of flexibility in MD simulations that typically look at distributions in only one or a few coordinates. The evaluation of a system’s energy from the force-field Hamiltonian is much more straightforward, subject to getting converged values and to all the approximations inherent in the force field or electronic-structure method used. To make EE methods faster and more practical, they often employ an implicit-solvent model to give a solvation free energy [18], as is done in the widely used Molecular Mechanics/Poisson–Boltzmann Surface Area (MM/PBSA) method and its Generalised-Born variant (MM/GBSA) [19–22]. In addition to the approximations in the solvation model, such as the choice of dielectric constant, surface or surface tension parameters, their application to explicit-solvent simulations brings about an inconsistency between the Hamiltonian used for sampling and that used for free-energy evaluation. A frequent approximation is to apply normal mode analysis to a minimised configuration under the assumption of a Gaussian distribution [19, 23], but this requires expensive matrix diagonalization for every minimum considered [24], and even then these minimized configurations are only approximately representative of thermalised ensembles. Consequently, the entropy contribution is often neglected [25], justified by the assumption that it is constant and therefore unimportant for relative binding calculations. A widely used method that does use thermalised ensembles is quasiharmonic analysis [26] based on coordinate covariance. Its assumption of a single Gaussian probability distribution permits a simple implementation, but this is known to over-estimate entropy [27, 28]. Alternative ways to use ensembles beyond the approximation is to integrate Boltzmann factors over minima numerically [28, 29], which is limited to a small number of minima, dihedral distributions [30], mutual information expansions [31] and the minimal spanning tree (MIST) variant [32], whose slowly converging nature limits their accuracy. For all non-Gaussian kinds of method, their classical formulation means they are limited to soft degrees of freedom, such as dihedrals or non-bonded interactions, and if applied to covalent bonds would give unphysically negative entropies, although one recent method includes dihedral correlations in a mutual-information manner supplemented by normal mode analysis [33, 34]. Moreover, as mentioned earlier, many methods use a continuum treatment of solvent. Treating the solutes and solvent differently leads to formulations that allocate the ideal-gas translational and rotational entropy to the binding molecules, which alters the understanding of the entropy loss of binding, with a larger proportion being assigned to the binding molecules and a corresponding entropy gain to the release of excluded-volume solvent [35]. Explicit solvent entropy has often been considered in binding, often to the exclusion of other entropic contributions and mostly in the context of inhomogeneous solvation theory [36–38]. Various other binding studies with combination terms have appeared such as molecular mechanics energy with the 3D Reference Interaction Site Model [39], continuum and explicit solvent [40], or inhomogeneous solvation theory with the loss of translational and rotational entropy [41] or with dihedral entropy [42].

**Fig. 1:**
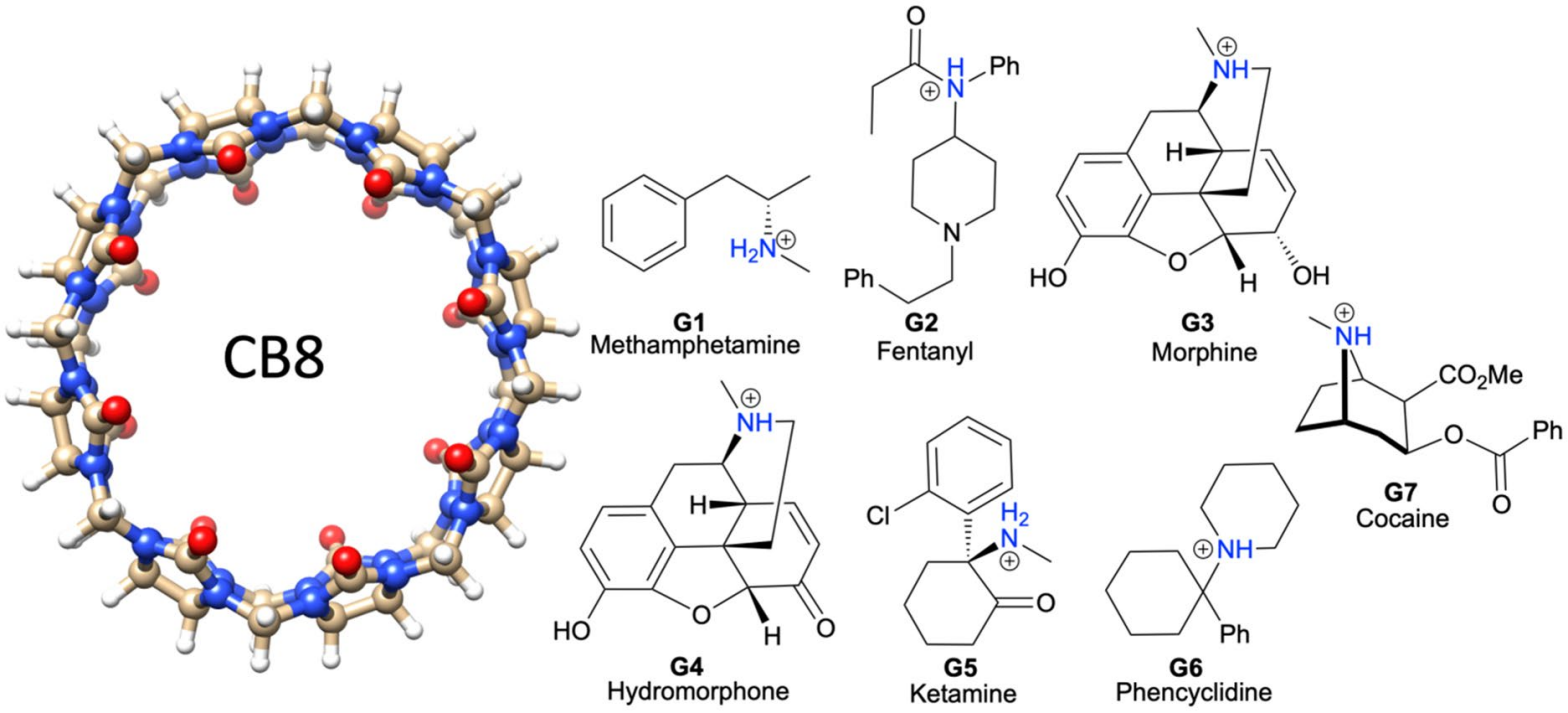
Chemical structures of the host CB8 and guests G1 to G7

To address the above-mentioned deficiencies of EE methods, we adapt the Multiscale Cell Correlation (MCC) method [43–45] to calculate the free energy of binding. MCC has been developed progressively, first in the context of cell theory for liquids [46, 47] and solutions [45, 48, 49], and later to account for correlations in flexible molecules [50], most recently at multiple length scales [43, 44, 51, 52]. A key feature of the theory is that it is applied to all molecules in the system in the same way, which makes it readily extendable to large flexible molecules in solution. We calculate the free energy of the unbound and bound molecules in water from the energy and entropy in molecular dynamics (MD) simulations, building off earlier work addressing the change in molecular rigid-body translational and rotational entropy of binding [25, 35]. We apply MCC to a series of host–guest complexes in the SAMPL8 “Drugs of Abuse” Blind Challenge (Statistical Assessment of the Modeling of Proteins and Ligands). Binding free energy has been a long-running quantity to calculate in the SAMPL Blind Challenges [53–56]. The SAMPL8 challenge involves the prediction of the binding free energies of seven drug molecules, illustrated in Fig. 1, to the drug-carrier molecule cucurbit[8]uril (CB8), whose binding free energies have been experimentally measured [57]﻿. As well as giving reasonable agreement with experiment binding free energy, MCC is able to explain these values by showing how the entropy change is distributed over all molecules in the system.

## Methods

### Free energy theory

**Fig. 2:**
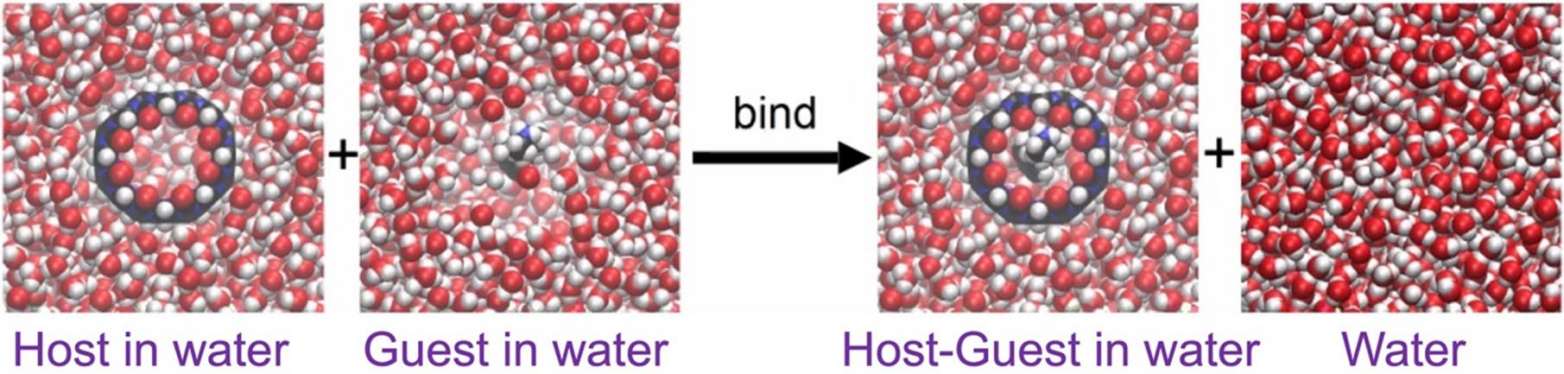
The four systems simulated to calculate the binding free energy by the EE method

The standard binding free energy (${\Updelta G}_{\text{bind}}^{\circ }$) of the host and guest molecules to form the host–guest complex in aqueous solution at the standard-state 1 M guest concentration is determined from the Gibbs free energies *G* calculated directly from simulations of the host in water, the guest in water, the host–guest complex in water, and bulk water:

1 $${\Updelta G}_{\text{bind}}^{\circ }=\left({G}_{\text{complex}}+{G}_{\text{water}}\right)-({G}_{\text{host}}+{G}_{\text{guest}}^{\circ })$$

as illustrated in Fig. 2. In energy–entropy methods, *G* is evaluated from the enthalpy *H* and entropy *S* using *G* = *H* − *TS* where *T* is temperature. The pressure–volume *PV* terms is omitted to allow the approximation *H* ≈ *E*, where *E* is the system energy, being small, on the order of 3 kJ mol^−1^ for the solutions studied here and even then almost entirely cancelling in the binding free energy difference.

In MCC, *S* is calculated in a multiscale fashion in terms of cells of correlated units. It is the sum of four different kinds of term ${S}_{ij}^{kl}$

2 $$S=\sum_{i}^{\text{molecule}}\sum _{j}^{\text{level}}\sum _{k}^{\text{motion}}\sum _{l}^{\text{minima}}{S}_{ij}^{kl}$$

First, *S* is partitioned over each kind of molecule *i*, whether host, guest or water. Second, for the molecules studied here, *S* has two levels of hierarchy *j*: molecule (M) and united atom (UA). Third, at each level, *S* is classified according to the type of motion *k*: translational or rotational. Fourth, for each motion, S is divided into vibrational and topographical terms *l*, which arise from the discretization of the potential energy surface into energy minima.

### Molecular entropy

An important feature of MCC is that the same entropy theory is applied to all molecules in the system. However, only the entropy of water molecules in the first hydration shell of the host and guest is considered here. This is because this reduces statistical noise that scales with the number of water molecules, and because the entropy of the remaining water molecules is assumed to change negligibly upon binding. Similarly, the entropy of the single neutralizing Cl^−^ ion is neglected in the calculations. To ensure balanced stoichiometry in binding, the number of bulk water molecules *N*_WB_ that contributes to *G*_water_ in Eq. 1 is chosen to ensure that

3 $${N}_{\text{WB}}+{N}_{\text{WS},\text{H}-\text{G}}={N}_{\text{WS},\text{H}}+{N}_{\text{WS},\text{G}}$$

where *N*_WS,H_, *N*_WS,G_, and *N*_WS,H–G_ are the number of water molecules around the host, guest and host–guest complex, respectively. Later, *N*_WB_ is partitioned into water released into bulk from the host or guest, *N*_WS,H–G_ is partitioned into water near the host or guest, and *N*_WS,H_ and *N*_WS,G_ are in turn partitioned into water released into bulk and water staying with the host or guest, respectively.

### Entropy for each level and motion

The axes of each molecule are taken as its principal axes with the origin at the molecular center of mass. All molecules considered here, treated as non-linear rigid bodies, have three translational and three rotational degrees of freedom. At the united-atom level, each united atom is defined as each heavy atom and its bonded hydrogen atoms. A united atom has three translational degrees of freedom and a number of rotational degrees of freedom depending on the number of hydrogens and resulting geometry: 3 for non-linear (> 1 hydrogen), 2 for linear (one hydrogen) and 0 for a point (no hydrogens). Its origin is taken as the heavy atom and the axes are defined with respect to the covalent bonds to the bonded hydrogens [43]. Note that it was necessary to use the reference frame of the host–guest complex when evaluating the entropy of the bound host at the united-atom level because this ensured a consistent alignment of the host with the guest.

### Vibrational entropy

The vibrational entropy, whether translational or rotational, or whether at the molecule or united-atom level, is evaluated in the harmonic approximation for the quantum hormonic oscillator:

4 $${S}^{\text{vib}}={k}_{\text{B}}\sum _{\text{i}=1}^{{N}_{\text{vib}}}\left(\frac{{hv}_{\text{i}}/{k}_{\text{B}}T}{{\text{e}}^{{hv}_{\text{i}}/{k}_{\text{B}}T}-1}-\text{ln}\left(1-{\text{e}}^{-{hv}_{\text{i}}/{k}_{\text{B}}T}\right)\right)$$

where ${k}_{\text{B}}$ is Boltzmann’s constant, ${N}_{\text{vib}}$ is the number of vibrations, *T* is temperature, *h* is Planck’s constant, and ${v}_{\text{i}}$ are the vibrational frequencies which are calculated from the eigenvalues λ_*i*_ of the appropriate covariance matrix

5 $${v}_{\text{i}}=\frac{1}{2\pi }\sqrt{\frac{{\lambda }_{\text{i}}}{{k}_{\text{B}}T}}$$

At the molecule level, *N*_vib_ = 6, corresponding to the *xyz* directions. Two covariance matrices are constructed, one from the mass-weighted forces for translation and one from the moment-of-inertia-weighted torques for rotation, each of these for the whole molecule with forces and torques halved in the mean-field approximation [43–47]. Their associated entropies are termed “transvibrational” and “rovibrational”. For transvibration at the united-atom level, *N*_vib_ = 3* N –* 6 where *N* is the number of united atoms in the molecule, and the six lowest-frequency motions have been removed to avoid duplication of transvibrational and rovibrational entropy at the molecular level. For rovibration at the united-atom level, *N*_vib_ is summed over the number of rotational degrees of freedom of each united atom. Covariance matrices are constructed as before but over all united atoms in the molecule and with halved torques in the mean-field approximation for weakly correlated degrees of freedom.

### Topographical entropy

For the topographical entropy at the molecular level, the translational term is known as the “positional” entropy and the rotational term is known as the “orientational” entropy. The positional entropy at the standard 1 M concentration is evaluated as

6 $${S}_{\text{M}}^{^\circ ,\text{transtopo}}\equiv {S}^{^\circ ,\text{pos}}={k}_{\text{B}}\ln\frac{1}{{x}_{\text{aq}}^{\circ }}$$

where ${x}_{\text{aq}}^{\circ }$ is the mole fraction of the molecule. For a solute when dilute, this is taken as 1/55.5, where 55.5 is the number of water molecules in the standard volume 1661 Å^3^, while for the solvent water ${x}_{\text{aq}}^{\circ } \approx 1.$ The orientational entropy for a molecule in solution is evaluated as

7 $${S}_{\text{M}}^{\text{rotopo}}\equiv {S}^{\text{or}}={k}_{\text{B}}\ln\frac{{N}_{\text{c}}^{(3/2)}{\pi }^{1/2}{p}_{\text{corr}}}{\sigma }$$

where *N*_c_ is the coordination number of the molecule, *S* is the symmetry number, and *p*_corr_ is the probability that the neighboring molecules are oriented suitably for each solute, *p*_corr_ = 1 while for water *p*_corr_ = 0.25 to account for hydrogen-bond correlation [43]. For a molecule in solution, *N*_c_ is the number of solvent molecules in the first hydration shell of the solute calculated using the Relative Angular Distance algorithm (RAD) [57]. For a guest bound to the CB8 host,

8 $${S}^{\text{or}}={k}_{\text{B}}\ln\frac{{\sigma }_{\text{host}}}{{\sigma }_{\text{guest}}}$$

where ${\sigma }_{\text{host}},$ the symmetry number of the CB8, equals 16, given its 8-fold and 2-fold rotational axes. At the united-atom level, the topographical entropy is known as the “conformational” entropy, with the translational term corresponding to dihedrals involving heavy atoms. It is calculated from the probability distribution of each set of unique conformations for all conformations having dihedrals of united atoms using

9 $${S}_{\text{UA}}^{\text{transtopo}}\equiv {S}^{\text{conf}}=-{k}_{\text{B}}\sum _{\text{i}=1}^{{N}_{\text{conf}}}{p}_{\text{i}}\ln{p}_{\text{i}}$$

where ${p}_{\text{i}}$ and *N*_conf_ are the probability and number of each set of conformations, respectively. Each conformation is defined adaptively whereby the dihedral is assigned to the nearest peak in the dihedral distribution calculated using a histogram with 30^◦^ bin widths [51]. The united-atom rotational topographical term is ignored because it corresponds to dihedrals involving exclusively hydrogens at one end and is either zero by symmetry, as in methyl groups, or small due to strong correlation with the solvent, as for hydroxyl groups. An additional entropic contribution to binding of − 0.5 *k*_B_ ln2 was included for guest G5 (Fig. 1) to account for the shift from half protonated when unbound to fully protonated when bound as pointed out in the SAMPL8 instructions.

### System preparation

The structural coordinates of the host and guest molecules were taken from the SAMPL8 Github website. All guests were built with their amino nitrogen in the protonated state; for guests G3, G4 and G7, the S stereochemistry was taken for G3 and G4 and the R stereochemistry for G7. The starting structure for the host–guest complex was taken as the lowest docked energy in the flexible docking of each guest molecule to the host using the AutoDock Vina software [58]. Amber Tools 19 [59] was used to create the topology and coordinate files of each system. The second-generation General AMBER Force Field (GAFF2) [60] with AM1-BCC partial charges as implemented in Antechamber [61] was used for the host and guest, TIP3P [62] was used for water, and the Joung and Cheatham parameters were used for the one chloride ion [63], which was added to neutralize the +1 charge of the guest. The entropic contribution of this chloride is not considered here, assuming the ion to be weakly interacting with the host and guest and therefore constant and canceling in the difference. This also justifies not modelling the exact experimental conditions [67] of 20 mM Na_2_HPO_4_, which would require force field parameters for the rarely modeled HPO_4_^2−^ ion and a system double the size to have the correct concentration. Four kinds of MD simulation were set up: (i) 1500 water molecules, (ii) the host molecules solvated in 1500 water molecules, (iii) each guest molecule in 1500 water molecules, and (iv) each host–guest complex in 1500 water molecules. All simulation boxes were cubic with side ~ 36 Å.

### Molecular dynamics simulation protocol

The simulations were performed with the GROMACS 2018.4 software package [64]. The topology and coordinate files for each system were converted from AMBER into GROMACS format using the GROMACS ParmEd tool because the entropy code used later does not yet work with AMBER trajectories. For equilibration, each system was minimized for 500 steps of steepest-descent minimization and heated gradually from 0 to 300 K for 100 ps of NVT molecular dynamics simulation using the V-rescale thermostat [65], followed by 100 ps of NPT simulation using the Parrinello-Rahman barostat [66] with a 2 ps time constant and the isothermal compressibility of water 4.5 × 10^− 5^ bar^−1^. The long-range electrostatic interactions were calculated using the Partial Mesh Ewald (PME) method with the Verlet cutoff-scheme, the non-bonded cutoff was 10 Å with periodic boundary conditions, and the time step was 2 fs. Data collection under the same conditions was run for 100 ns of MD simulation, with forces and coordinates saved every 100 ps to give 1000 frames for analysis. Entropies were calculated using MCC [44, 45] with additional terms for binding [48]. Calculation of all entropy terms was performed with two separate python codes, one code for the solutes (https://github.com/arghya90/CodeEntropy) and an in-house code for the solvent, each reading in the force, coordinate and topology files for each simulation. Four simulations were needed for each binding calculation as shown in Fig. 2 and each MD simulation was run in triplicate with slightly different starting structures, yielding $\Updelta G$ of binding via Eq. 1.

### Error analysis

The standard error of the mean (SEM) for *G*, *H* and *S* are calculated from the standard deviation of the values from those derived from the three separate simulations

10 $$\text{SEM}= \frac{\sigma }{\sqrt{n}}$$

where *n* = 3 is the number of simulations. The mean average error (MAE) with respect to experiment is

11 $$\text{MAE}=\frac{\sum \left|\Updelta G-\Updelta {G}_{\text{expt}}\right|}{n}$$

where *n* = 7 is the number of molecules.

## Results and discussion

**Fig. 3:**
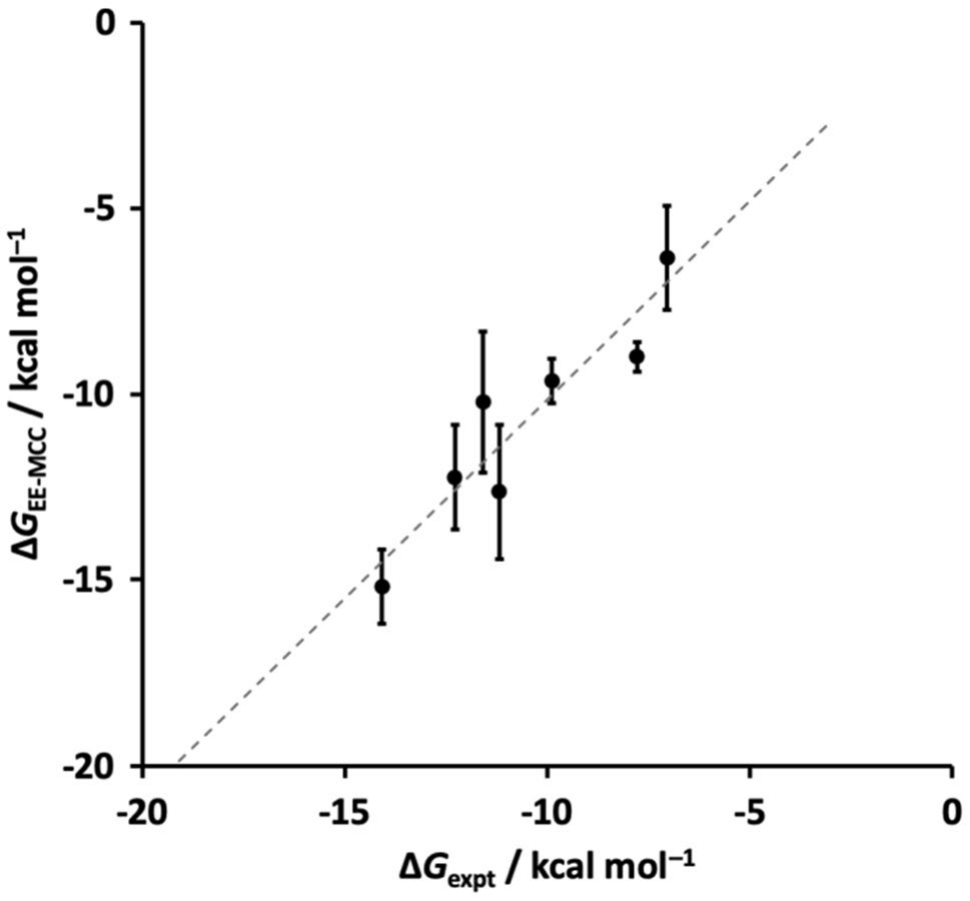
EE-MCC Gibbs free energies of binding (error bars are the SEM) versus experiment [67]

The calculated binding Gibbs free energies together with SEM error bars are plotted in Fig. 3 versus experiment [67]. The values of the EE-MCC and experimental [67] binding Gibbs free energies are listed in Table 1, together with the Δ*H* and *T*Δ*S* components.﻿ The MAE for the Δ*G* averaged unsigned error over all molecules is 0.9 kcal mol^−1^ and for Δ*H* and *T*Δ*S* they are 2.0 and 1.8 kcal mol^−1^, respectively. Evidently, there is some correlation between the enthalpy and entropy that brings about a lower error in the binding Gibbs free energy than in these two components, particularly for compounds G2, G4, G5 and G7 which have larger but compensating errors in Δ*H* and *T*Δ*S*. Plots to indicate the convergence of the simulation energy versus time are given in Fig. S1, together with their gradients in Table S1.

**Table 1:** Predicted binding free energies, enthalpies and entropies versus experiment [67]

Guest	Δ*G* (kcal mol^−1^)		Δ*H* (kcal mol^−1^)		*T*Δ*S* (kcal mol^−1^)	
	EE-MCC	Expt	EE-MCC	Expt	EE-MCC	Expt
G1	− 6.3 ± 1.4	− 7.1	− 7.6 ± 0.1	− 7.8	– 1.3 ± 1.4	– 0.8
G2	− 9.6 ± 0.6	− 9.9	− 5.0 ± 0.7	– 10.8	4.6 ± 0.9	– 0.9
G3	– 10.2 ± 1.9	– 11.6	– 11.9 ± 0.2	– 13.6	– 1.7 ± 1.7	– 2.0
G4	– 12.6 ± 1.8	– 11.2	– 11.7 ± 0.4	– 15.8	1.0 ± 1.5	– 4.6
G5	– 12.2 ± 1.4	– 12.3	– 14.0 ± 0.02	– 17.3	– 1.7 ± 1.4	– 5.0
G6	– 15.3 ± 1.0	– 14.1	– 14.4 ± 0.1	– 14.9	1.0 ± 1.0	– 0.8
G7	− 9.0 ± 0.4	− 7.9	– 11.5 ± 0.3	− 8.3	− 2.5 ± 0.1	– 0.3

## Entropy components with MCC

**Fig. 4:**
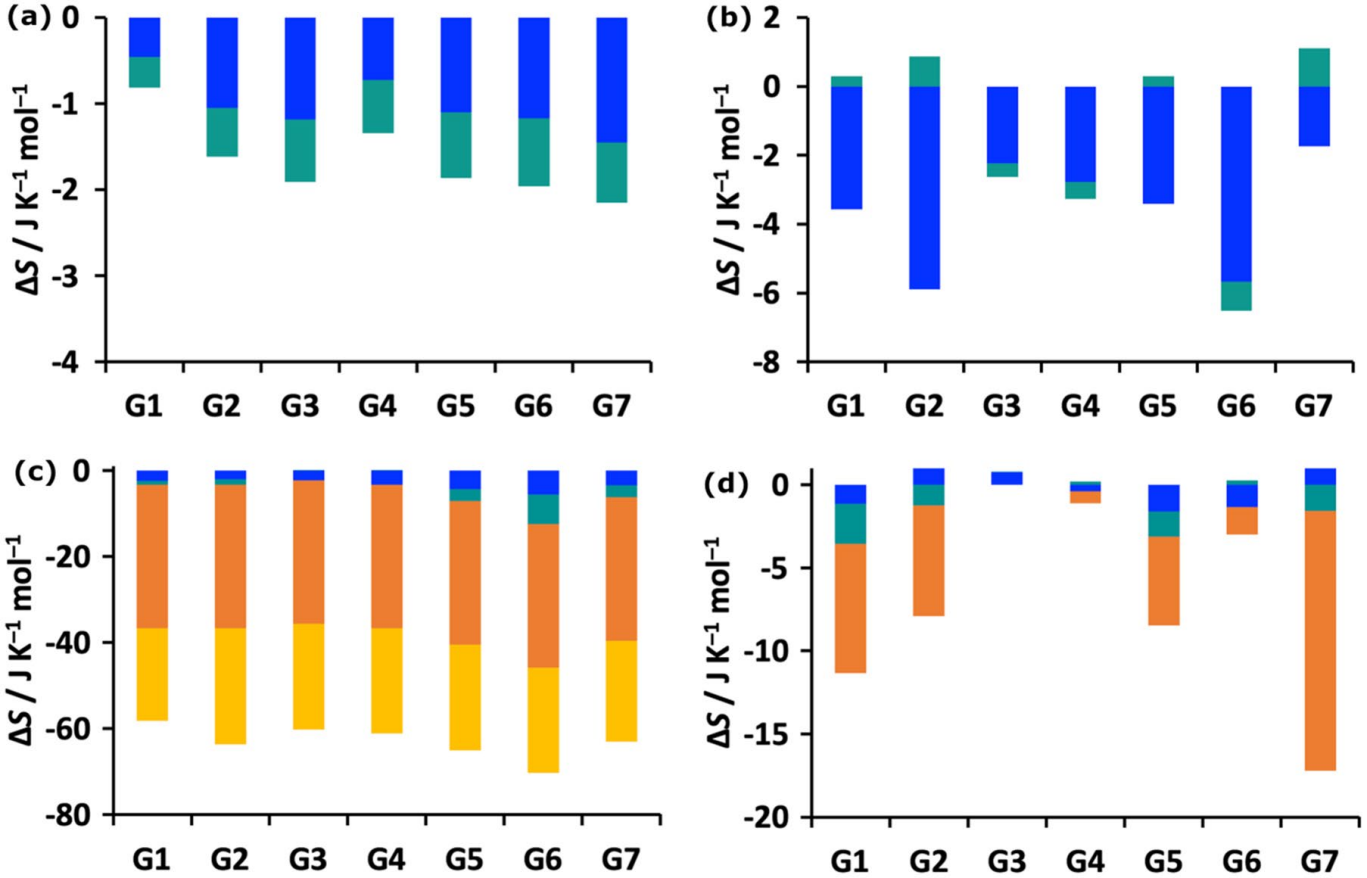
Binding entropy components for the **a** host at molecular level, **b** host at united-atom level, **c** guest at molecular level, and **d** guest at united-atom level. The components are transvibrational (blue), rovibrational (turquoise), positional/conformational (orange), and orientational (yellow)

MCC yields the entropy of the system and its decomposition over molecules, level, motion and minima according to Eq. 2. In Fig. 4 we show plots for the changes in vibrational and topographical entropy components upon binding for the host and guest at molecule and united-atom levels of hierarchy. The corresponding SEMs of these components are given in Table S2. The host entropy, which is all vibrational, decreases for all guests but only by a small amount. The contributions are slightly larger at the united-atom level and the rovibrational term is sometimes weakly positive. The positional and orientational entropy of the host is taken not to change, given that it defines the reference frame for the binding process. The decrease in entropy of the guest is much larger because it comprises the loss of positional and orientational entropy, the former constant for all guests at 1 M concentration and the latter dependent on the size of the molecule via the number of first-shell water molecules. There is a smaller but moderate decrease in conformational entropy of up to 15 J K^−1^ mol^−1^ for the more flexible guests G7, G1, G2 and G5 which have more freely rotating dihedrals. The guests have only a small decrease in vibrational entropy, as for the host, with the occasional tiny increase at the united-atom level. The changes in dihedral profiles for the flexible guests (numbered in Fig. S2) upon binding can be seen in Figs. S3–S7. This shows a general narrowing in distributions when bound that is consistent over all three simulations. There is some variance for G2 and G6, which brings about a SEM in guest conformational entropy of 5.2 J K^−1^ mol^−1^ which corresponds to just below half a kcal mol^−1^ in *T*Δ*S*. The total guest entropy losses of 60–75 J K^−1^ mol^−1^ are similar to the values of 71–73 J K^−1^ mol^−1^ from an earlier study on protein–ligand systems with comparatively sized ligands that only considered the molecule-level entropy [35].

**Fig. 5:**
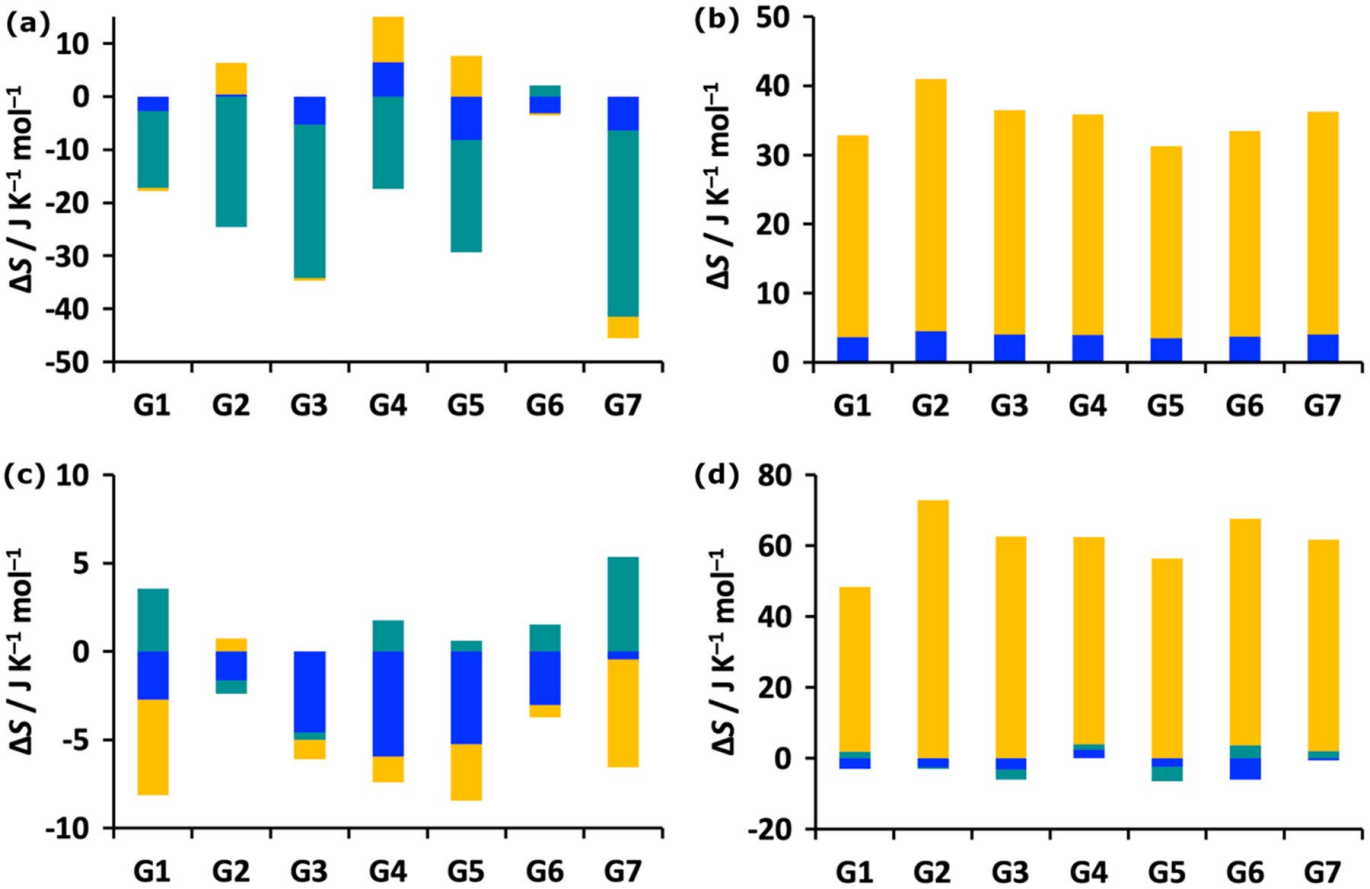
Changes in binding entropy components for the **a** water staying in the hydration shell of the host (WS), **b** water released from the host into bulk water (WB), **c** water staying in the hydration shell of the guest (WS), and **d** water released from the guest into bulk water (WB). Coloring is as in Fig. 4

The corresponding changes in water entropy are shown in Fig. 5, with the SEMs provided in Table S2. There is a fairly sizeable decrease in rovibrational entropy for water around the host upon binding, with the exception of G6 which has a slight increase, possibly because its cationic nitrogen is fully buried inside the host and so cannot constrain water molecules. The changes in the transvibrational and orientational entropy of water are smaller and either higher or lower, depending on the guest. The changes in water hydrating the guest are smaller, given that the guest has little solvent exposure when bound; in most cases the decrease is transvibrational or orientational, with some increase in rovibrational. For water released into bulk from either host or guest, there is a large gain in orientational entropy for all guests, consistent with the larger number of hydrogen-bonding neighbours of a water molecule in bulk. There is a larger contribution from water around the guest because the guest becomes more buried and releases more water molecules. Water released from the host is seen to gain a small amount of transvibrational entropy, while the vibrational terms change little for the guest. The component SEMs in Table S2 show that the largest contribution to the error in entropy comes from water staying with the host, which is understandable because that relates to the most flexible and largest number of atoms, involving on the order of 80 water molecules. For similar reasons, the next largest SEM is from water that is released from the guest.

The corresponding entropy components for all contributing species when unbound or bound are shown in Tables 2 and 3, together with the number of contributing water molecules, either staying bound in the hydration shell of the host or guest (WS) or being released into bulk (WB).These numbers are consistent with the trends in Figs. 4 and 5. Their most insightful revelation is the magnitudes of the entropies involved. Clearly, most of the entropy is in the solvent water, and the size of this entropy term scales near linearly with the number of water molecules in the first hydration shell. The contributions from the host and guest molecules for their respective unbound cases are much smaller at only about 14 % and 14–20 %, respectively. Most of the host entropy, 85 %, is at the united atom level, and of that, 80 % is transvibrational and the rest rovibrational while at the molecule level these two terms are comparable in size, as seen in earlier work [34, 43, 44, 48]. For the guest the two levels have similar amounts of entropy, depending on the size of the ligand and at the 1 M concentration being used here. The numbers of water molecules in each of the four categories makes clear that the guest is almost entirely desolvated upon binding and that the host loses comparatively fewer water molecules to accommodate the guest, supporting the finding in Fig. 4 that guest desolvation contributes more than host desolvation for the systems studied here.

**﻿Table 2:** Entropy components of unbound and bound host and associated water (J K^−1^ mol^−1^)

	H	H-G1	H-G2	H-G3	H-G4	H-G5	H-G6	H-G7
${S}_{\text{H},\text{M}}^{\text{transvib}}$	70	70	69	69	69	68	69	69
${S}_{\text{H},\text{M}}^{\text{rovib}}$	74	73	73	73	73	73	74	73
${S}_{\text{H},\text{UA}}^{\text{transvib}}$	662	659	656	660	659	661	658	658
${S}_{\text{H},\text{UA}}^{\text{rovib}}$	159	159	160	158	158	159	158	160
${S}_{\text{H}}^{\text{conf}}$	0	0	0	0	0	0	0	0
${S}_{\text{WS}}^{\text{transvib}}$	4079	3566	3440	3505	3526	3583	3555	3507
${S}_{\text{WS}}^{\text{rovib}}$	1515	1310	1253	1275	1290	1312	1232	1270
${S}_{\text{WS}}^{\text{or}}$	251	648	632	638	652	661	647	635
*N*_WS_	87.4	76.4	73.7	75.2	75.4	76.9	76.2	75.3
${S}_{\text{WB}}^{\text{transvib}}$		516	644	573	563	491	525	569
${S}_{\text{WB}}^{\text{rovib}}$		190	238	211	208	181	194	210
${S}_{\text{WB}}^{\text{or}}$		123	153	136	134	117	125	135
*N*_WB_		11.0	13.7	12.2	12.0	10.5	11.2	12.1

**Table 3:** Entropy components of unbound and bound guests and associated water (J K^−1^ mol^−1^)

System	Component	G1	G2	G3	G4	G5	G6	G7
Unbound guest	${S}_{\text{G},\text{M}}^{\text{transvib}}$	62	68	66	67	66	67	68
	${S}_{\text{G},\text{M}}^{\text{rovib}}$	59	67	61	62	65	68	64
	${S}_{\text{G}}^{\text{pos}}$	33	33	33	33	33	33	33
	${S}_{\text{G}}^{\text{or}}$	45	50	48	48	46	48	49
	${S}_{\text{G},\text{UA}}^{\text{transvib}}$	41	137	96	95	84	81	119
	${S}_{\text{G},\text{UA}}^{\text{rovib}}$	74	118	86	87	72	99	96
	${S}_{\text{G}}^{\text{conf}}$	20	29	0	2	16	7	17
	${S}_{\text{WS}}^{\text{transvib}}$	1144	1786	1458	1460	1335	1496	1663
	${S}_{\text{WS}}^{\text{rovib}}$	419	658	540	538	496	545	610
	${S}_{\text{WS}}^{\text{or}}$	72	126	97	278	254	277	311
	*N*_WS_	24.3	37.9	30.9	31.1	28.4	31.7	35.4
Bound guest	${S}_{\text{G},\text{M}}^{\text{transvib}}$	60	66	64	64	62	63	65
	${S}_{\text{G},\text{M}}^{\text{rovib}}$	58	64	61	61	62	62	62
	${S}_{\text{G}}^{\text{pos}}$	0	0	0	0	0	0	0
	${S}_{\text{G}}^{\text{or}}$	23	23	23	23	23	23	23
	${S}_{\text{G},\text{UA}}^{\text{transvib}}$	40	138	96	95	82	80	121
	${S}_{\text{G},\text{UA}}^{\text{rovib}}$	72	116	86	84	70	99	95
	${S}_{\text{G}}^{\text{conf}}$	12	22	0	1	10	6	1
	${S}_{\text{WB}}^{\text{transvib}}$	1031	1395	1239	1242	1201	1248	1190
	${S}_{\text{WB}}^{\text{rovib}}$	377	514	459	458	446	454	437
	${S}_{\text{WB}}^{\text{or}}$	198	258	231	236	228	231	223
	*N*_WB_	21.9	29.6	26.3	26.5	25.5	26.4	25.3
	${S}_{\text{WS}}^{\text{transvib}}$	110	390	214	212	129	245	472
	${S}_{\text{WS}}^{\text{rovib}}$	45	144	81	82	51	92	179
	${S}_{\text{WS}}^{\text{or}}$	16	73	40	40	22	45	82
	*N*_WS_	2.4	8.3	4.7	4.6	2.9	5.3	10.1

## Conclusions

A new energy–entropy method called EE-MCC has been presented to calculate the free energy of binding and applied to a series of aqueous host–guest complexes in the SAMPL8 “Drugs of Abuse” Blind Challenge. EE-MCC accounts for the entropy of all flexible degrees of freedom of the system in a consistent and general manner. The calculated binding Gibbs free energy values are in good agreement with experimental results having average standard error of mean 0.9 kcal mol^−1^. The main feature of MCC is that it provides the entropy components over all molecules and all degrees of freedom in the system at a hierarchy of length scales. There is a large loss of positional and orientational entropy that is fairly similar for all guests, with the orientational entropy loss larger for larger guests. There is a smaller loss of conformational entropy, depending on the flexibility of the guest. There are also smaller decreases in vibrational entropy of the host, guest and contacting water. These losses are compensated by a large gain in orientational entropy of water released to bulk, with the larger contribution coming from water that was hydrating the guest.

## Electronic Supplementary Material

Below is the link to the electronic supplementary material.

Supplementary Material 1 (PDF 5200 kb)
