# Supplementary material for: Energy–entropy method using multiscale cell correlation to calculate binding free energies in the SAMPL8 host–guest challenge
Source: J Comput Aided Mol Des. 2021 Jul 15;35(8):911–21. doi: 10.1007/s10822-021-00406-5 (PMC8367938; doi:10.1007/s10822-021-00406-5)
Supplement: Supplementary file 1 — Supplementary Material 1 (PDF 5200 kb) [file 10822_2021_406_MOESM1_ESM.pdf]

Supplementary Information

Energy-Entropy Method Using Multiscale Cell Correlation to Calculate Binding Free Energies in the SAMPL8 Host-Guest Challenge

Hafiz Saqib Ali[1,2], Arghya Chakravorty[4], Jas Kalayan[1,2], Samuel P. de Visser[1,3] and Richard H. Henchman*[1,2]

[1] Manchester Institute of Biotechnology, The University of Manchester, 131 Princess Street, Manchester M1 7DN, United Kingdom
[2] Department of Chemistry, The University of Manchester, Oxford Road, Manchester M13 9PL, United Kingdom
[3] Department of Chemical Engineering and Analytical Science, The University of Manchester, Oxford Road, Manchester M13 9PL, United Kingdom
[4] Department of Chemistry, University of Michigan, Ann Arbor, Michigan 48109, United States

*Corresponding Author: Richard H. Henchman

rhen7213@uni.sydney.edu.au
Present Address: Sydney Medical School, The University of Sydney, Sydney, 2006 NSW, Australia

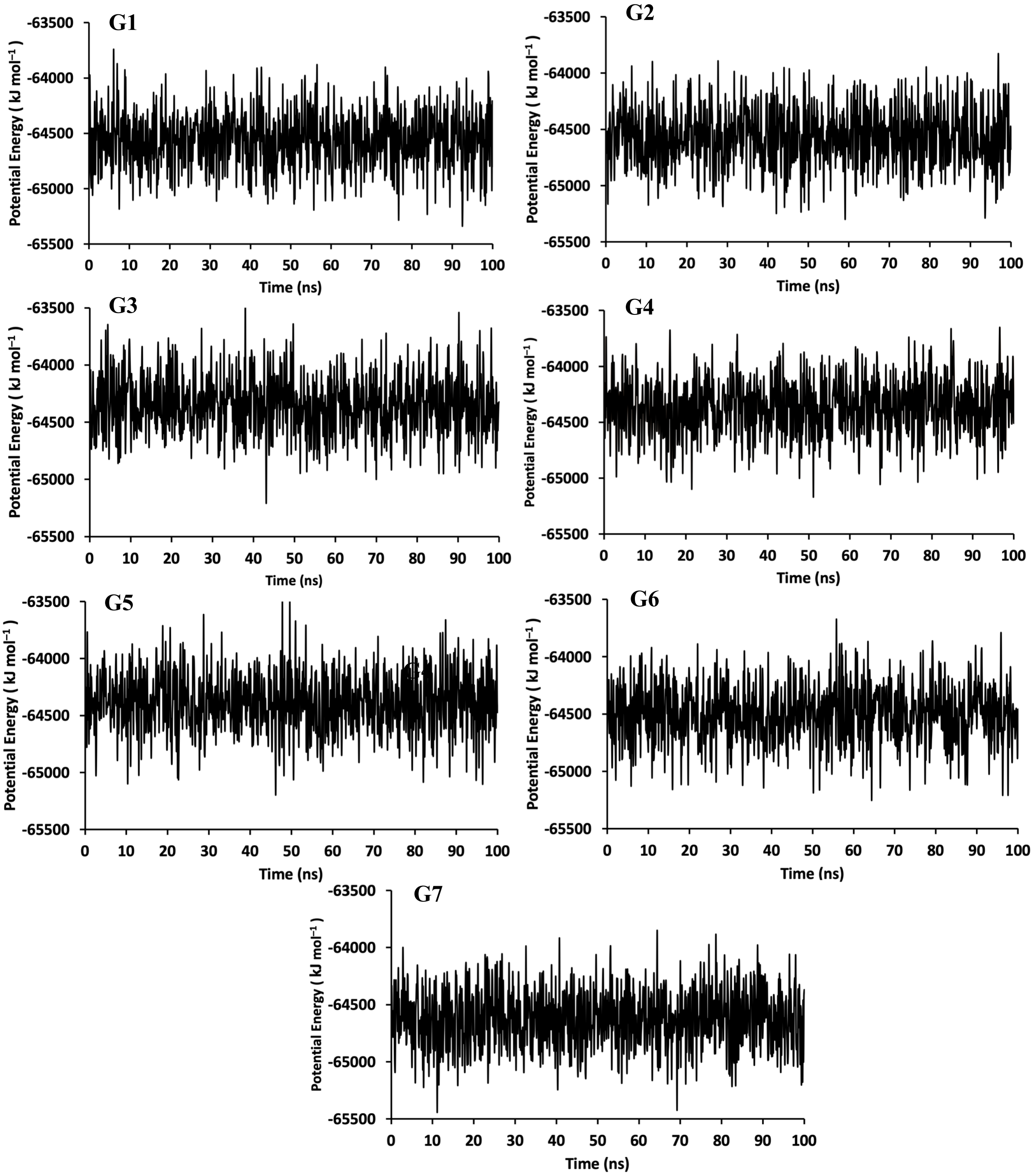

Figure S1. Potential energy versus time for the host-guest systems G1 to G7.

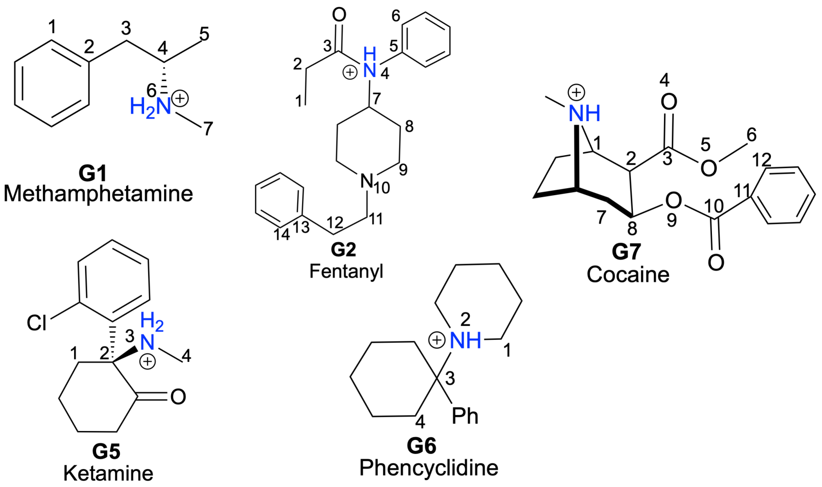

Figure S2. Numbering of the flexible dihedrals in guests: G1, G2, G5, G6 and G7.

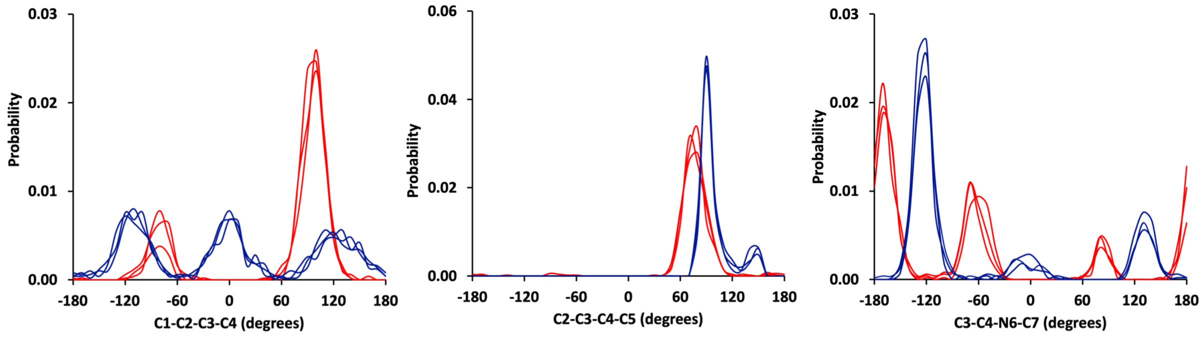

Figure S3. Dihedral distributions for all flexible dihedrals in guest G1 in the three unbound simulations (blue) and three bound simulations (red).

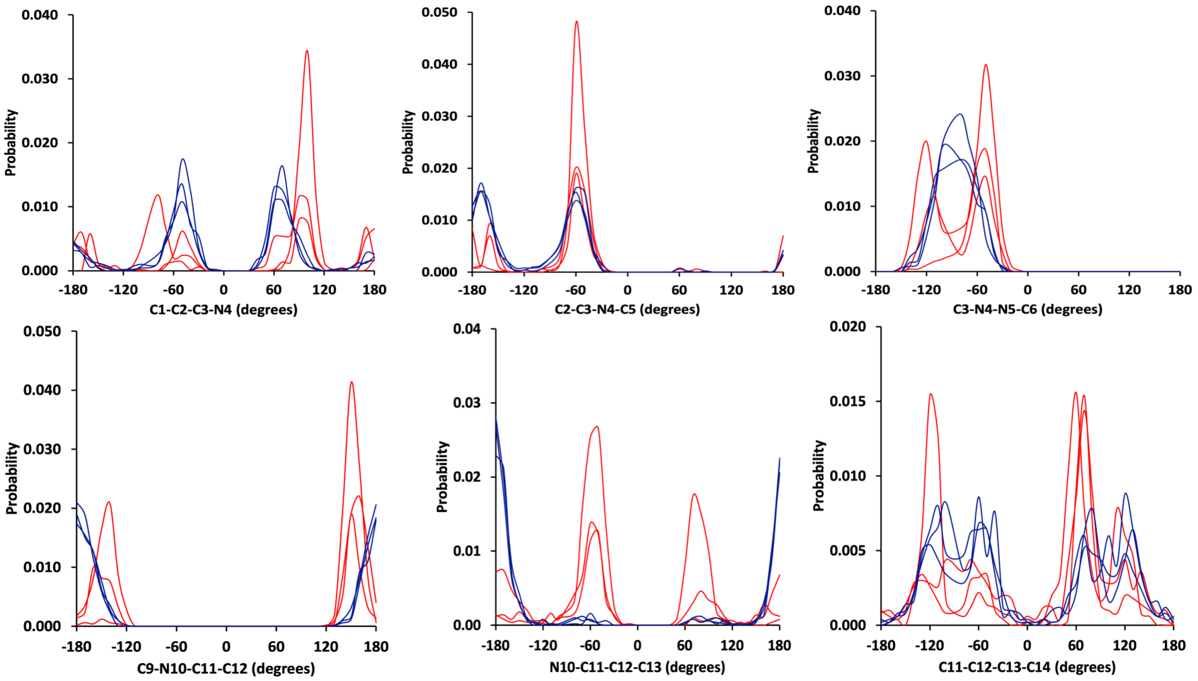

Figure S4. Dihedral distributions for all flexible dihedrals in G2 as in Figure S3.

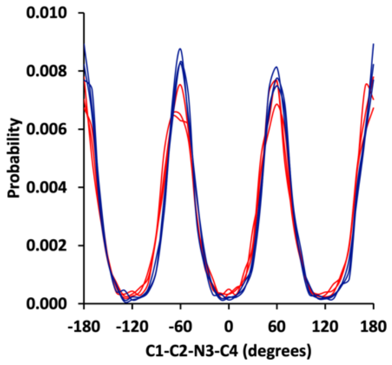

Figure S5. Dihedral distributions for all flexible dihedrals in G5 as in Figure S3.

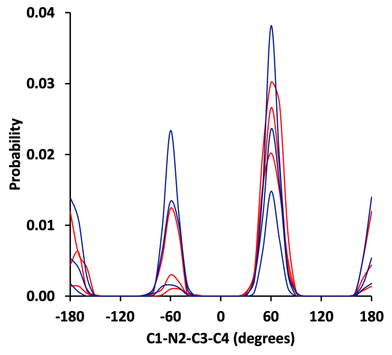

Figure S6. Dihedral distributions for all flexible dihedrals in G6 as in Figure S3.

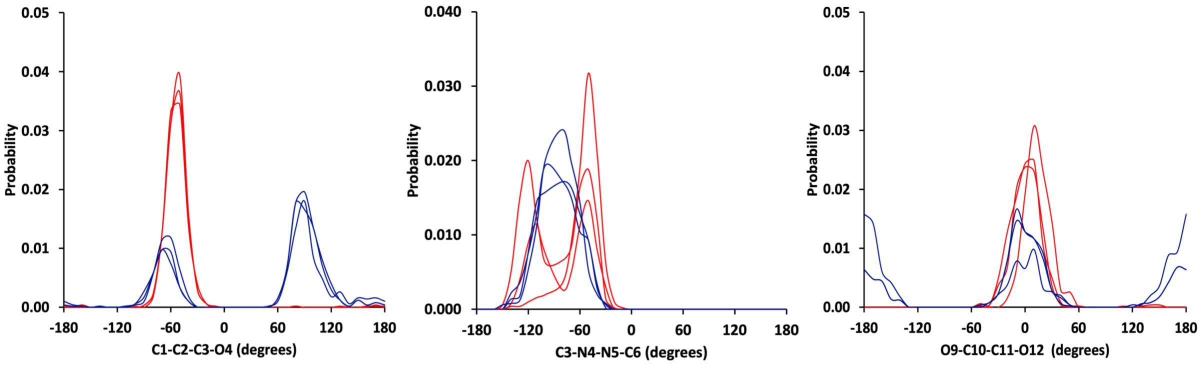

Figure S7. Dihedral distributions for all flexible dihedrals in G7 as in Figure S3.

Table S1. Slopes of the Lines of Best Fit (kJ mol^{-1} ns^{-1}) for the Potential Energy versus Simulation Time in Figure S1

Set of Calculations	G1	G2	G3	G4	G5	G6	G7
1st	0.003	0.139	–0.398	0.389	–0.138	0.111	0.142
2nd	0.026	–0.019	–0.025	0.019	–0.013	0.182	–0.243
3rd	0.501	–0.165	–0.408	0.328	0.014	–0.308	0.328

Table S2. Standard Error of the Mean (SEM) for the Components of the Entropy Differences (J K^{-1} mol^{-1}) in Figures 4 and 5

System	G1	G2	G3	G4	G5	G6	G7
$\Delta S_{\mathrm{H,UA}}$	1.8	0.4	3.4	1.0	1.0	1.2	1.7
$\Delta S_{\mathrm{H,M}}$	0.5	0.6	0.4	0.8	0.2	0.6	0.7
$\Delta S_{\mathrm{G,UA}}$	0.6	0.9	1.6	0.8	0.8	5.2	0.7
$\Delta S_{\mathrm{G,M}}$	0.2	0.9	0.8	0.5	0.5	0.5	0.5
$\Delta S_{\mathrm{H,WS}}$	15.0	4.3	12.2	7.2	7.8	10.3	6.4
$\Delta S_{\mathrm{H,WB}}$	5.5	2.0	0.9	0.4	1.0	0.6	1.4
$\Delta S_{\mathrm{G,WS}}$	3.1	4.5	1.0	4.2	2.2	3.3	1.0
$\Delta S_{\mathrm{G,WB}}$	1.1	7.7	6.4	5.6	2.9	5.0	2.4
